# Scanning electrochemical microscopy and its potential for studying biofilms and antimicrobial coatings

**DOI:** 10.1007/s00216-020-02782-7

**Published:** 2020-07-21

**Authors:** Giada Caniglia, Christine Kranz

**Affiliations:** Institute of Analytical and Bioanalytical Chemistry, Ulm University, Albert-Einstein-Allee, 11, 89081 Ulm, Germany

**Keywords:** Scanning electrochemical microscopy, Biofilm, Bacteria, Quorum sensing, Antimicrobial

## Abstract

Biofilms are known to be well-organized microbial communities embedded in an extracellular polymeric matrix, which supplies bacterial protection against external stressors. Biofilms are widespread and diverse, and despite the considerable large number of publications and efforts reported regarding composition, structure and cell-to-cell communication within biofilms in the last decades, the mechanisms of biofilm formation, the interaction and communication between bacteria are still not fully understood. This knowledge is required to understand why biofilms form and how we can combat them or how we can take advantage of these sessile communities, e.g. in biofuel cells. Therefore, in situ and real-time monitoring of nutrients, metabolites and quorum sensing molecules is of high importance, which may help to fill that knowledge gap. This review focuses on the potential of scanning electrochemical microscopy (SECM) as a versatile method for in situ studies providing temporal and lateral resolution in order to elucidate cell-to-cell communication, microbial metabolism and antimicrobial impact, e.g. of antimicrobial coatings through the study of electrochemical active molecules. Given the complexity and diversity of biofilms, challenges and limitations will be also discussed.

## Introduction

The term biofilm was used in technical and environmental microbiology already for a long time to describe bacterial sessile aggregates as a cause of biofouling [1]; however, Costerton et al. [2] first introduced the term biofilm in biomedical research, studying the proteomic of *Pseudomonas aeruginosa* microcolonies. The authors described them as an interconnected and well-organized community of bacteria, able to stick to both biotic and abiotic surfaces, exhibiting increased antimicrobial resistance in comparison with planktonic cell cultures. It is estimated that bacteria in biofilms become up to 1000 times more resistant to antimicrobial agents [3]. Due to the elevated resilience, biofilms affect our societies in many ways ranging from health-related issues, such as contamination in medical devices, e.g. urinary catheters [4], cardiovascular devices [5] and orthopaedic prosthetics [6], in food industries [7], agriculture [8] and biocorrosion and microfouling in sewer pipes, shipping industries, etc. [9–11]. Biofilms are the cause of about 65% of chronic diseases in humans [12]. For example, *Staphylococcus aureus* is able to colonize the upper respiratory tracts and cause chronic diseases such as rhinitis and bronchial asthma [13]. *Salmonella* [14], *Pseudomonas* [15], *Bacillus* [16] and *Listeria* [17] are biofilm-forming food pathogens, causing the food industry enormous costs each year [18]. *Xylella fastidiosa*, a plant pathogenic bacterial species, which causes huge economic losses in crops (grapevine, *citrus*, etc.) mostly in the Americas, has reached Europe causing severe damage to olive-growing regions in Italy [19]. *X. fastidiosa* is known to obstruct the water pathways within more than 100 different plants after irreversible adhesion to the xylem surface and formation of biofilms. Not all biofilms are harmful, and biofilms are used in microbial fuel cell (MFC) technology, converting chemical energy from water-based organic matter into electricity. Besides, some bacterial strains contribute to bioremediation processes, e.g. oil-consuming bacteria [20] and microbes that can uptake heavy metals [21].

In order to design new and effective strategies to prevent biofilm formation, understand antimicrobial resistance, improve performances of MFCs, etc., it is important to understand the biochemical processes during the microbial colonization and the biofilm formation [22]. Biofilm formation is a complex multistage process [2] which starts with the adhesion of the bacteria at a surface. After an irreversible attachment to the surface, followed by cell division and proliferation, microbes start to secrete extracellular polymeric substances (EPSs) composed of proteins, polysaccharides and nucleic acids. EPS is a viscous matrix that promotes the cohesion of cells and provides physical protection to bacteria [23, 24]. During the colonization, bacteria release products, such as metabolites, which are related to the development and reproductive activities of the microbes. Identifying and mapping their concentration gradients is important to understand biofilm behaviour. Given the heterogeneity of biofilms [25], metabolite secretion of cells may be different depending on the location in the biofilm, due to the internal gradients of e.g. nutrients, oxygen and pH [26].

**Fig. 1:**
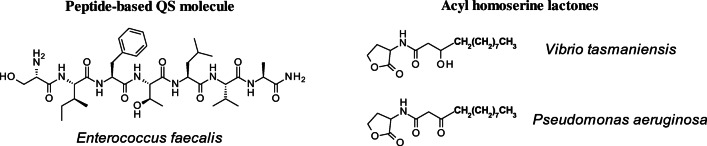
Characteristic QS molecules and their chemical structures

One of the most important mechanisms coordinating the biofilm formation is quorum sensing (QS) [27]. Biofilms exhibit advanced cell-to-cell communication governed by the secretion of small specific molecules, which regulates an interaction among cells through induced gene expression. The nature of QS molecules depends on the type of bacteria. As shown in Fig. 1, peptide-based QS molecules are usually expressed by Gram-positive species, such as *Enterococcus* [22], while acyl-homoserine lactone (AHL) derivates are characteristic of Gram-negative bacteria, like *Vibrio* [28] and *Pseudomonas* [29]. These molecules can change the gene transcription profile, activating or repressing specific QS-controlled genes [30]. Gene mutation, during the first stages of biofilm formation, regulates the biogenesis of the flagella called type I pili, which are not only essential to the first interaction with the surface but also contribute to the pathogenicity of the bacteria [31, 32]. Inhibiting the secretion of such molecules is one strategy to avoid the formation of biofilms. However, this requires an understanding of how bacteria communicate, and as a diverse group of chemical species is produced, their identification is important. Figure 1 represents characteristic QS molecules for Gram-positive and Gram-negative bacteria.

**Fig. 2:**
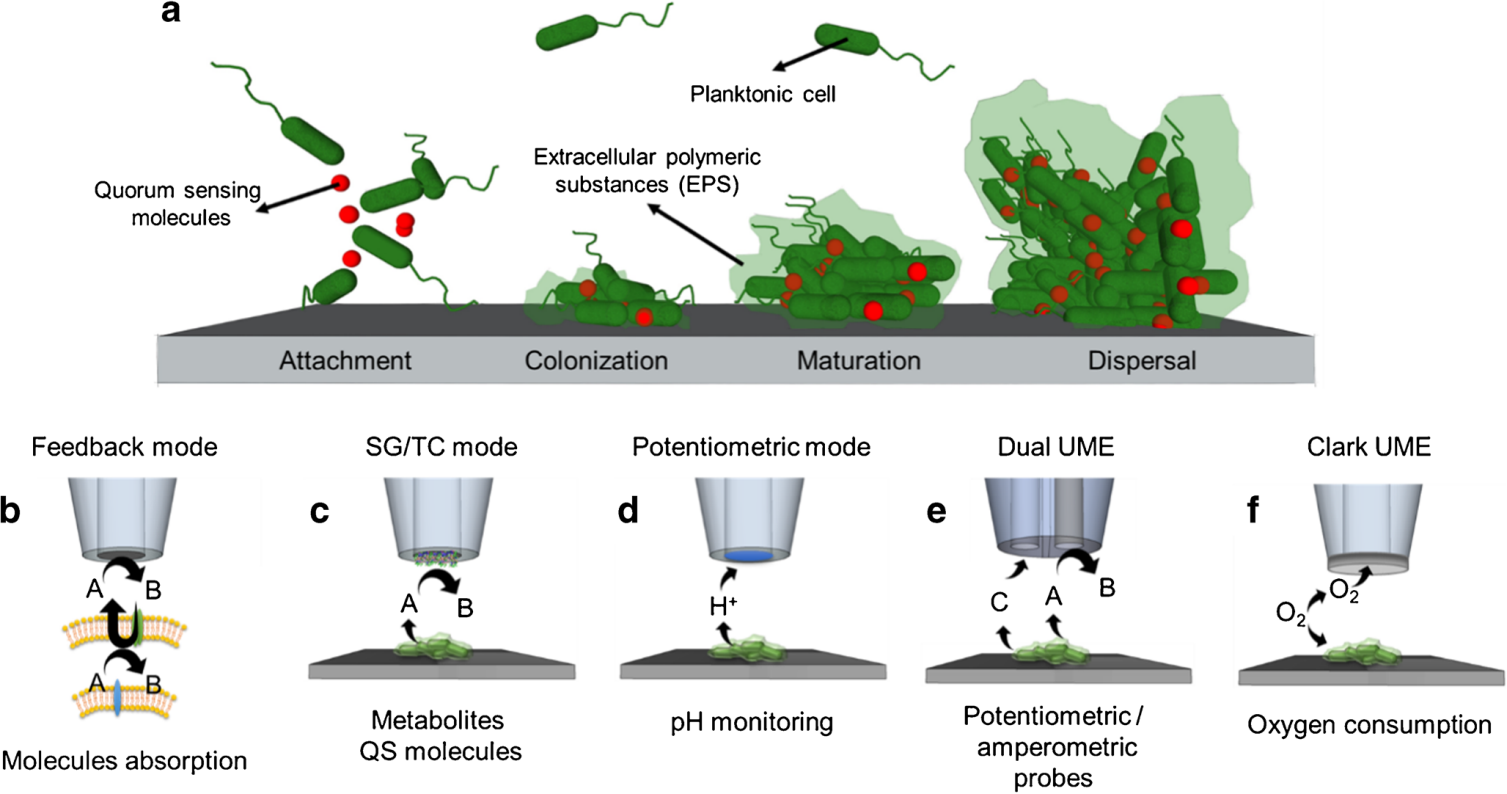
(**a**) Schematic of multistage biofilm formation. (**b**–**f**) SECM modes used to study biofilms

In order to understand multistage processes involved in biofilm formation (as depicted in Fig. 2a), a multitude of strategies has been developed and many different methods are employed, ranging from advanced microscopic and spectroscopic techniques [33, 34] to mass spectrometry [35, 36] and proteomic methods [37, 38]. The interested reader is directed to recent reviews on methodical approaches for more information [39–41].

The adhesion of bacteria at surfaces, which is the initial step of biofilm formation, has been intensively studied via atomic force microscopy (AFM) [42] for Gram-negative and Gram-positive bacteria including e.g. *Xylella fastidiosa* [43]. An AFM-derived method termed “single-cell AFM force spectroscopy” (SCFM) [44] allows mapping of adhesion properties at the single cell level, i.e. the study of the cell-cell and cell-surface interactions in different environments and types of surfaces [45–47]. The role of microbial adhesins and bacterial pili, as well as their molecular binding mechanism, has been studied by SCFM [48, 49].

Real-time advanced optical techniques such as confocal laser scanning microscopy (CLSM) contributed to the knowledge of the spatial structure of biofilms with single-cell-level resolution and the quantitative determination of structural parameters, such as roughness, biovolume and thickness using highly specific fluorescent probes [50]. The combination of CLSM with fluorescence in situ hybridization (FISH) allowed the spatial identification of bacteria in mixed communities such as multibacterial oral biofilms [51, 52]. Coupling CLSM with the fluorescence lectin-binding analysis (FLBA) has opened up new possibilities for in situ determination of glycoconjugate and its distribution via 3D imaging, which is highly interesting as glycoconjugates are one of the species present in the EPS produced by bacteria during biofilm formation [53–55]. Time-based monitoring of EPS compounds was also demonstrated with non-destructive, label-free techniques that do not require e.g. fluorophores, such as infrared attenuated total reflection (IR-ATR) spectroscopy [56, 57] or Raman microscopy [58, 59]. IR-ATR spectroscopy not only provides biochemical and physiological information of the biofilm but also allows monitoring how biofilms evolve over time by the changes of the IR-ATR fingerprint region [60]. Besides biofilm composition, also the antimicrobial effects of biofilm inhibitors have been studied via IR-ATR [56].

Although intensive research efforts have been dedicated towards understanding the formation and the growth of biofilms, to date, there are still open questions such as “which mechanism governs the formation and stability of biofilms?” This review is focused on microelectrochemical approaches, mainly scanning electrochemical microscopy (SECM) and its potential for studying key chemical parameters that are influencing biofilm formation and growth.

### Microelectrochemistry

Electrochemical methods, such as potentiometry, voltammetry or electrochemical impedance spectroscopy (EIS), have been used in biofilm research, for example, in respect to non-destructive monitoring of bacterial communities [61], the investigation of redox-active bacteria such as *Shewanella oneidensis* MR-1 [62] and *Geobacter sulfurreducens* [63], the effect of electrode material to minimize biofouling [64] and for insight into quorum sensing [65]. For example, electrochemical approaches have been used to study long-distance electron transport at *Geobacter sulfurreducens* that can use electron acceptors residing outside the cell for respiration [63]. In particular, microelectrochemistry and SECM are attractive methods for real-time investigation of redox-active small molecules with micron-scale resolution. Studies on effects of polarized substrates and extracellular electron transfer play a significant role in biofuel cell research. In respect to microelectrochemical studies and SECM investigations on biofilm formation and cellular communication, the effect of substrate potential or the effect of depletion of molecules such as oxygen has been little addressed in the literature.

Electrochemical microsensors and microbiosensors for the analysis of biofilms have been employed for more than 30 years [66] to study micron-scale chemical gradients and metabolism in microbial communities. The advantage of using microsensors is associated with the improved sensitivity and selectivity. Depending on the molecule of interest, various types of microsensors have been developed, some are also commercially available. The most common potentiometric microsensors are ion-selective microelectrodes using liquid-ion-exchange membranes for i.e. pH [67], ammonium and nitrite [68]. Oxygen, nitrogen-containing molecules and hydrogen peroxide, which are correlated with respiration, nitrogen cycles and oxidative stress of bacteria, respectively, are also mapped with amperometric micro(bio)sensors [69, 70]. Another interesting approach in terms of miniaturized electrochemical sensing is related to integrated circuits using complementary metal-oxide-semiconductor (CMOS) technology. For example, spatially resolved imaging of three electroactive phenazine metabolites of *Pseudomonas aeruginosa* PA14 biofilms was obtained using a high-density array with 1824 gold integrated electrodes multiplexed to 38 parallel output channels [71].

To date, only a few reviews are available, addressing, among other topics, the versatility and advantages of SECM in biofilm research [72, 73]. This review will give an overview on microelectrochemical approaches, focusing on SECM to study important factors, such as nutrients, metabolites, ion concentrations profiles and QS at bacterial communities, and also addressing current challenges and possible limitations. For detailed information on SECM, the readers are directed towards some excellent reviews, providing a detailed description of imaging modes and applications as well as some fundamental aspects of SECM [72–76].

### Scanning electrochemical microscopy

In SECM, miniaturized electrodes (currently electrodes with radii ranging from 25 μm down to several nm) [77, 78] are used as SECM tips to map information of the sample surface, while the tip is moved across the sample surface. In dependence of the SECM mode, the surface morphology and the tip size, the current response may depend on both topography and electrochemical activity [74].To date, biofilm studies have been performed with conventional SECM, where the SECM tip is positioned via recording *z*-distance vs. current curves (approach curves) and then either perform stationary measurements or scan the SECM tip in a constant height across the sample surface. In feedback mode SECM (Fig. 2b), electron transfer (ET) reactions within bacterial cells can be investigated by using a hydrophilic redox mediator, which can cross the outer cell membrane and behave as an electron acceptor in the respiratory chain of the microbial cell [79]. In this context, the mediator regenerates in the periplasm of the cell and an increase of the current response is registered. The rate constant of the mediator regeneration, knowing its concentration and formal potential, can provide valuable information about the electron transport mechanism in the respiration chain.

Gradients of key parameters such as pH, oxygen, redox potential, virulence factors, metabolites and ions evolve at biofilms, controlled by diffusional processes through the 3D architecture of the EPS matrix [80]. Besides, it is known that bacterial metabolism, associated with the production of acidic by-products, leads to pH changes within the biofilm, which may differ from external pH values. Substrate generation/tip collection (SG/TC) (Fig. 2c) belongs to most relevant SECM modes for studying bacteria and biofilms, since redox species released from the cells are collected by the SECM probe, whose electrochemical response reveals the spatial and temporal concentration profile of the species. Concentration profiles and kinetics of target molecules, such as metabolites, QS molecules or drugs consumed, which are relevant in the different stages of the biofilm formation, have been demonstrated in SG/TC mode [81, 82]. The potentiometric mode of SECM allows to study label-free changes in a concentration of molecules or species that are not electroactive by using ion-selective microelectrodes (see Fig. 2d). Although pH changes are frequently studied with fluorescence probes as spatiotemporal resolution can be achieved [83, 84], potentiometric probes for pH measurements such as Sb/SbO_2_ [85], Ir/IrO_*x*_ [86], polyaniline [87] and ultramicroelectrodes (UME) and carbon-based pH microsensors [88] are attractive as no labelling is required. Also, dual ultramicroelectrodes (Fig. 2e), which allow both potentiometric and amperometric measurements to obtain morphological information and pH profiles with the same probe, have been shown. Oxygen consumption (Fig. 2f) can be studied using a Clark UME [89], which consists of a platinum ultramicroelectrode with a silver ring, acting as a reference/counter electrode.

## Applications in biofilm studies

While electrochemical methods have been used in microbial studies for quite some time, SECM has only been employed more frequently in microbial research within the last decade. Gram-positive and Gram-negative bacteria such as *Staphylococcus aureus* [90], *Rhodobacter sphaeroides* [79], *Salmonella typhimurium* [91], *Pseudomonas aeruginosa* [92, 93], *Vibrio fischeri* [94], *Streptococcus gordonii* [95] and *Escherichia coli* [82, 96–98] have been investigated with SECM. Earlier SECM studies were mainly focused on mapping oxygen consumption of microbial cells [99]. Also, the transmembrane charge transfer in *Rhodobacter sphaeroides* using hydrophilic and hydrophobic redox mediators in SECM feedback mode has been investigated [79]. The study revealed that hydrophobic redox species can penetrate both the outer cell membrane and the cytoplasmatic membrane, while hydrophilic mediator only crosses the outer membrane.

**Fig. 3:**
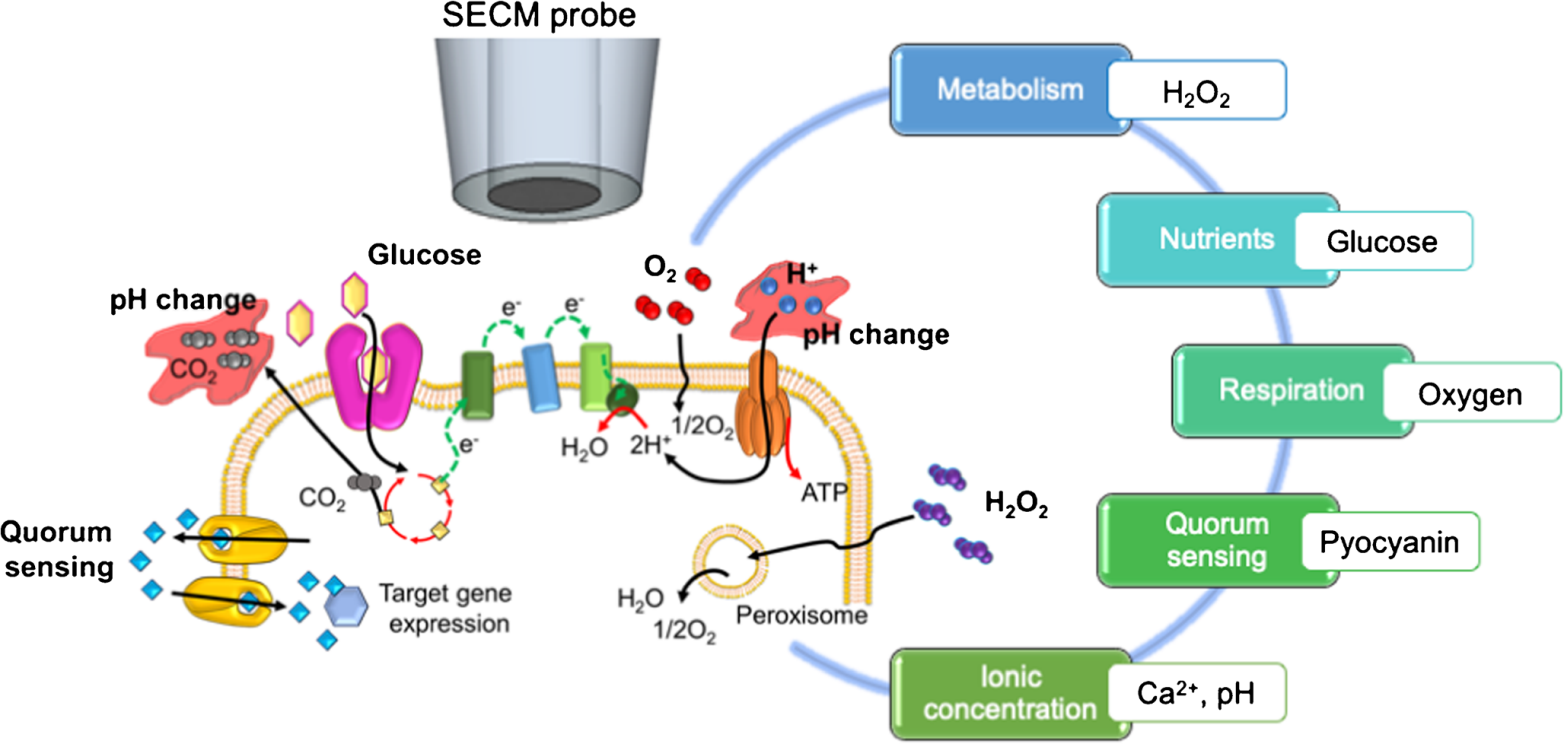
Representative parameters in biofilm research investigated with SECM

In recent years, applications of SECM regarding biofilms have been focused on the study of key parameters such as electroactive metabolites, QS molecules, consumption of nutrients and oxygen, ion concentrations and pH (Fig. 3).

Hydrogen peroxide (H_2_O_2_) is produced by several aerobic bacteria associated with the human body such as *Streptococcus*, *Pneumococcus* and *Lactobacillus* strains. H_2_O_2_ is responsible, among other reactive oxygen species (ROS), for oxidative stress and plays a significant role in inflammation processes. How microbial H_2_O_2_ influences host-microbial interactions is, to date, still not fully understood [100]. SECM was used to detect H_2_O_2_ at biofilms to study glucose metabolism and catalase activity during the formation of *Streptococcus gordonii* [95] and *Vibrio fischeri* [94] biofilms. To date, most studies used spectrophotometric methods for H_2_O_2_ determination using a peroxidase assay following a protocol by Gilliland [101]. However, H_2_O_2_ can be detected electrochemically at either bare microelectrodes, using enzyme-based microsensors [102], or electrocatalytically modified microelectrodes such as Prussian blue [103] or platinum black [104], which allow to map H_2_O_2_ concentrations down to the nanomolar range.

Liu et al. [81] used the SG/TC mode (Fig. 2c) to map H_2_O_2_ concentration above the Gram-positive bacterium *Streptococcus gordonii* and co-cultured biofilms of *S. gordonii* and *Aggregatibacter actinomycetemcomitans*, a Gram-negative bacterium. *S. gordonii* converts sugars into lactic acid, producing H_2_O_2_ in the presence of O_2_. A higher H_2_O_2_ level was detected in case of *S. gordonii* biofilm, compared to its planktonic counterpart. The authors could also show that *A. actinomycetemcomitans* co-cultured with *S. gordonii* reduces H_2_O_2_ levels due to the presence of a protein (KatA) of the catalysed enzyme family. Line scans clearly revealed a decrease in H_2_O_2_ levels above the *A. actinomycetemcomitans* spot in comparison to increased levels above a KatA-deficient *A. actinomycetemcomitans* mutant. The recorded H_2_O_2_ levels in the vicinity of the biofilms determined by SECM were much higher (mM range) compared to fluorescence-based H_2_O_2_ determination in the supernatant solution assay. Abucayon et al. [94] investigated the catalase activity of *Vibrio fischeri* biofilms. Catalase is able to produce oxygen as a by-product of the oxidation of H_2_O_2_ in a process called disproportionation, as a defence mechanism of the cells against oxidative stress. SECM measurements revealed that the level of H_2_O_2_ is related with the catalase activity, which changes with the time of incubation of the biofilm, in comparison with the planktonic bacteria. Theoretical modelling along the experiments predicted that the disproportionation of H_2_O_2_ to form H_2_O and O_2_ was 3 × 10^6^ molecule of H_2_O_2_ per bacterium and second. The high activity of catalase e.g. allows bacteria to survive under the oxidative stress produced by the host against the colonization. To improve the detection limit of H_2_O_2_ in biological measurements, a platinum UME was modified with a multiwalled carbon nanotube-Pt nanoparticle-ionic liquid–based composite (Pt-MWCNT-IL), which achieved a linear range from 250 nM to 7 mM and a sensitivity three times higher than that of other metal-based SECM probes [95]. The authors investigated the metabolic activity in the presence of glucose of *S. gordonii* biofilms by using a dual-Pt/Pt-MWCNT-IL sensor and the effect on the H_2_O_2_ profile in simulated human saliva conditions, i.e. adding lactoperoxidase (LPO) and potassium thiocyanate (SCN^−^) as H_2_O_2_-decomposing agents. Preliminary measurements showed that the presence of LPO and thiocyanate (SCN^−^) decreases the concentration of the H_2_O_2_. Thus, it could be assumed that the effects of oxidative stress on the oral cavity can be alleviated by the presence of LPO and SCN^−^.

**Fig. 4:**
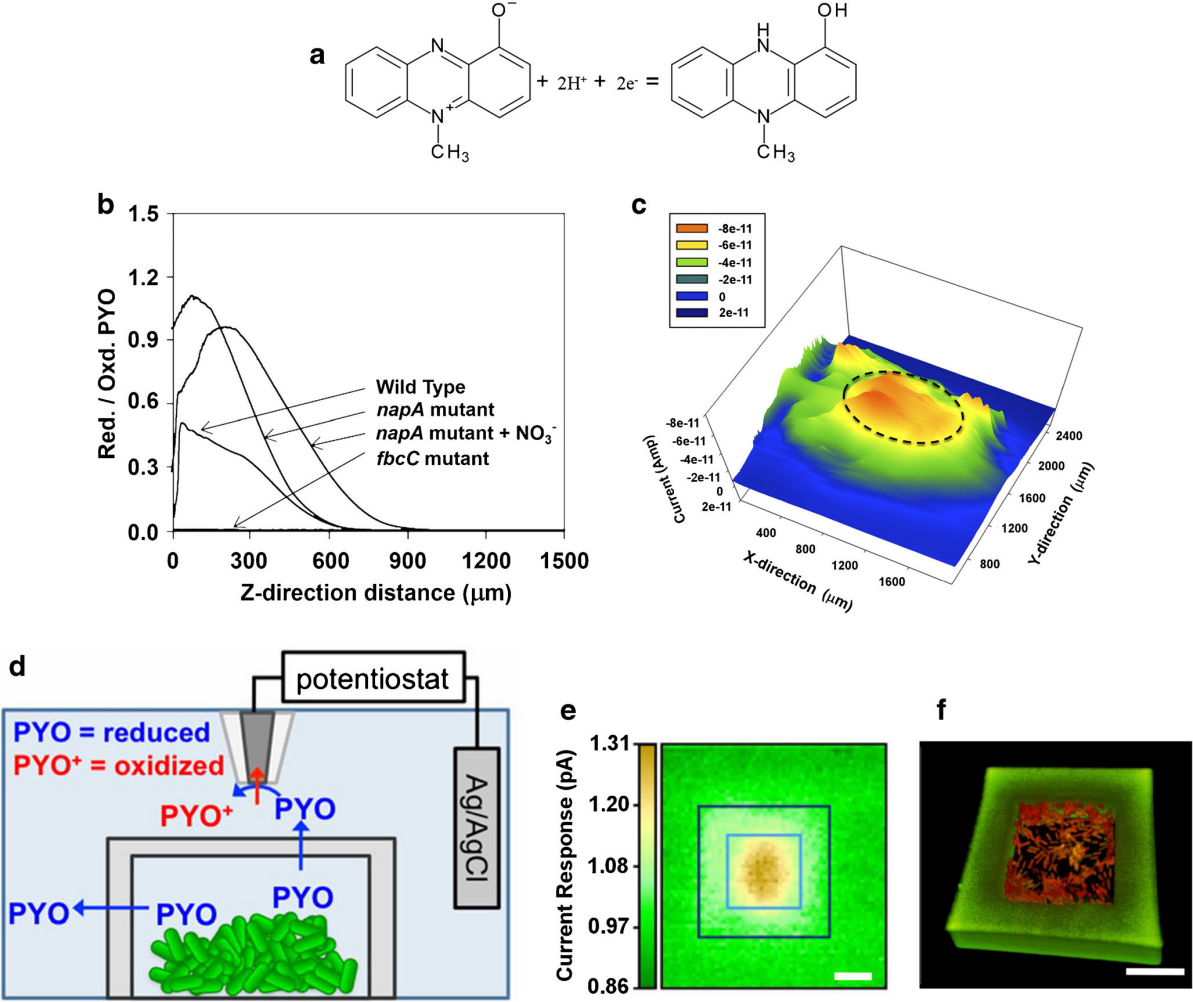
(**a**) Redox reaction of pyocyanin. (**b**) The *z*-direction reduced pyocyanin (PYO) profiles above *P. aeruginosa* napA and fbcC mutants. (**c**) A constant height SECM image of a *P. aeruginosa* biofilm. *E*_tip_ = − 0.3 vs. Ag/AgCl to oxidize PYO. Dotted line indicates the position of the biofilm. (**a**–**c**) Reproduced from Koley et al. [93], with permission from the National Academy of Sciences (copyright 2011). (**d**) Schematic of the microtrap-SECM system for measuring PYO in real time. (**e**) SECM image of PYO response collected above a microtrap containing more than 500 WT *P. aeruginosa*. SECM tip potential of 0 V vs. Ag/AgCl to oxidize the pyocyanin. (**f**) Three-dimensional confocal reconstruction that shows ~ 700 cells (red) in the microtrap (walls appear green). (**a**–**c**) Reproduced from Connell et al. [92], with permission from the National Academy of Sciences (copyright 2014)

SECM enables to determine in situ the local concentration of redox species with temporal and spatial resolution, which is highly attractive to quantitatively determine label-free redox-active QS molecules. It is known that the activation of QS is concentration-dependent of the signalling molecules secreted by the bacteria and depends on the microbial population density [27]. In a collaborative effort, Connell et al. [92] and Koley et al. [93] used SECM to study quorum sensing at a bronchial human pathogenic bacterium *Pseudomonas aeruginosa*. Pyocyanin (PYO), a redox-active secondary metabolite (see Fig. 4a) secreted by *P. aeruginosa*, was studied in respect to the concentration profile of its reduced form above *P. aeruginosa* as high concentrations of this electroactive molecule are actively maintained by the bacteria. PYO is an important metabolite not only suppressing other microbes but also maintaining redox homeostasis and regulating biofilms in case of nutrient depletion [105]. The concentration gradient of reduced PYO, which is termed electrocline that extends up to several hundred microns into solution, was investigated in the presence of other electron acceptors like nitrate (NO_3_^−^) and in the presence of Fe^3+^ that actively re-oxidize PYO as shown in Fig. 4b. The authors also demonstrated the capability of SECM to image the concentration of PYO above *P. aeruginosa* as shown in Fig. 4c. In another SECM study [92], the authors investigated the distance of bacterial colonies trapped in 3D-printed cages as shown in Fig. 4d. Two mutant strains of *P. aeruginosa* (PYO-producing strain and QS-responsive strain) were placed in microtraps separated by an 8-μm-thick wall. Aggregates with approx. 500 cells were sufficient to induce QS-mediated communication between the two cages. Figure 4e and f show a SECM image reflecting the concentration of PYO above a trap containing wild-type *P. aeruginosa* and the 3D confocal re-construction for counting the cells, respectively.

A linear range limit of detection for PYO in SG/TC mode was reported with 2–120 μM, although no limit of quantification (LOQ) was given. Hence, it might be useful in future studies to consider whether the cell number-pyocyanin ratio is really due to the minimum concentration that has been produced by the bacteria or is defined by the limit of quantification of the electrochemical method.

**Fig. 5:**
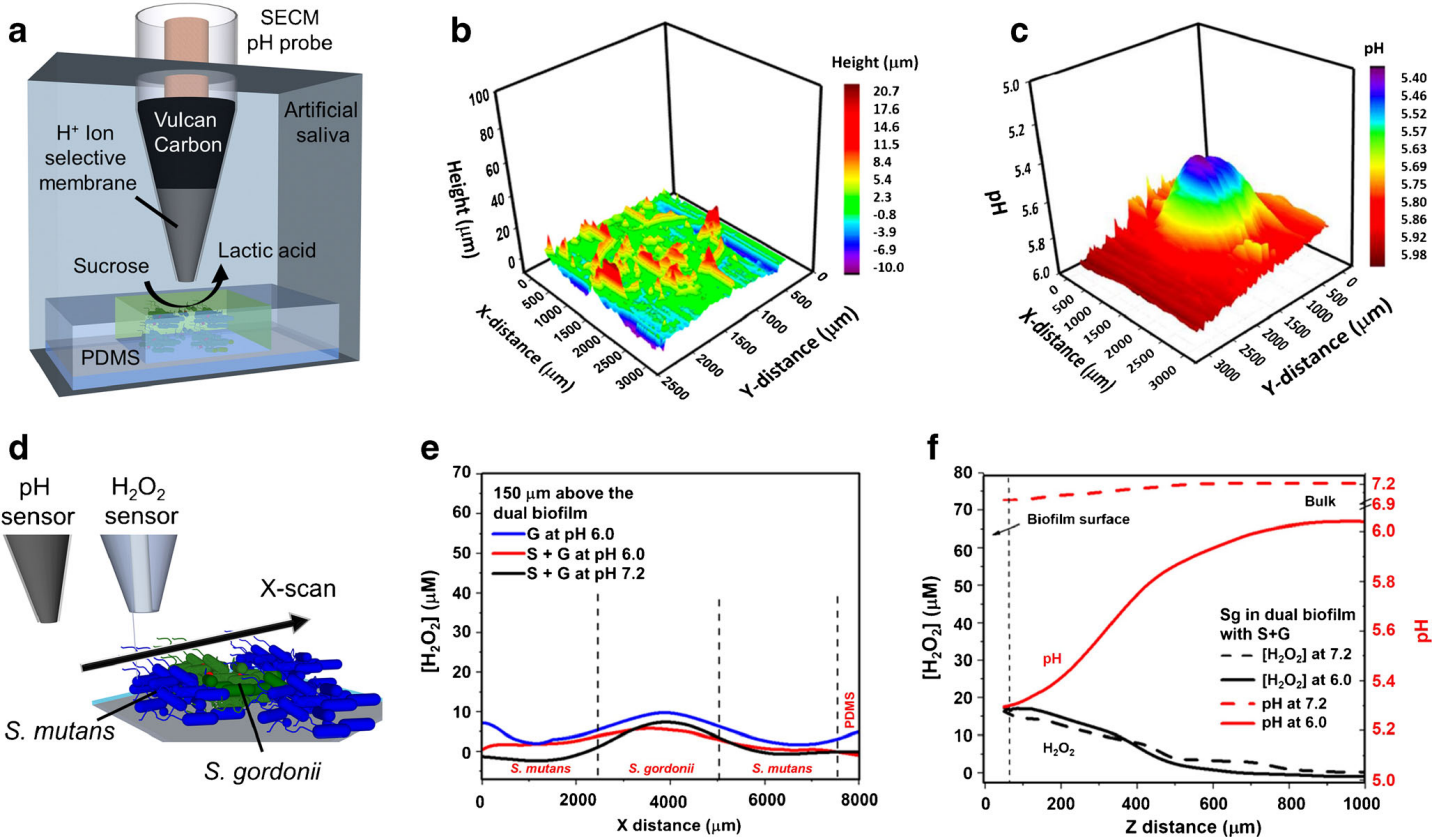
(**a**) Schematic of the *S. mutans* bacterial gel biofilm substrate and the pH microprobe used in SECM experiments. (**b**) Three-dimensional morphological image of the *S. mutans* bacterial gel biofilm substrate recorded 60 μm above the biofilm with the pH microprobe (scan speed, 30 μm/s) in 1 mM ferrocenemethanol in artificial saliva (pH 7.2) at 23 °C. (**c**) Three-dimensional pH image recorded 60 μm above the biofilm with the pH microprobe (scan speed, 30 μm/s) after the addition of 30 mM sucrose in artificial saliva (pH 6.0) at 37 °C. (**d**) Scheme of dual recording using a pH microsensor and a H_2_O_2_ microsensor. (**e**) *x*-direction H_2_O_2_ profile 150 μm above the dual bacterial biofilm in the presence of glucose (G) and glucose + sucrose (S) at pH 6.0 and 7.2. (**d**) *z*-direction H_2_O_2_ and pH profile from 50 μm above *S. gordonii* in the dual bacterial biofilm to 1000 μm above in the bulk solution in the presence of G + S at pH 6.0 (solid lines) and 7.2 (dashed lines). (**b**), (**c**), (**e**), (**f**) Reprinted from Joshi et al. [88], with permission from the American Chemical Society (copyright 2017)

Joshi et al. [88] and Ummadi et al. [106] recently presented potentiometric microsensors that could be also used for amperometric measurements (e.g. for positioning the microsensor in SECM experiments) by mixing ionophores with conductive carbon materials. For example, the group used a Ca^2+^ ion-selective microelectrode in combination with a dual microelectrode consisting of a bare Pt and a Pt-polyaniline pH microsensor in combination with SECM, mostly in stationary experiments, to monitor the pH and Ca^2+^ ion concentration profiles during the calcification process of *Sporosarcina pasteurii* biofilms. *S. pasteurii* is known to form calcite in the presence of urea and Ca^2+^ ions [107], hydrolyzing urea in brine solution, which results in an increase in local pH that causes the precipitation of calcite. The same group mapped with the carbon-based pH microsensor topography and pH profiles of pathogenic *Streptococcus mutans* biofilms, which belong to acid-producing bacteria as shown in Fig. 5a–c [88]. The authors also studied the metabolic interplay between the H_2_O_2_-producing *S. gordonii* and *S. mutans* organized in an alginate gel (Fig. 5d) in artificial saliva. In dependence of the buffer capacity of the artificial saliva, a gradual decrease in H_2_O_2_ concentration was observed over time accompanied by an acidification (decrease in pH value) as shown in Fig. 5e and f. Recently, the same group studied glucose uptake by *S. mutans* with a microbiosensor based on glucose oxidase immobilized on functionalized multiwalled carbon nanotubes (f-MWCNTs) and 1-butyl-4-methyl-pyridinium hexafluorophosphate mixtures [108].

**Fig. 6:**
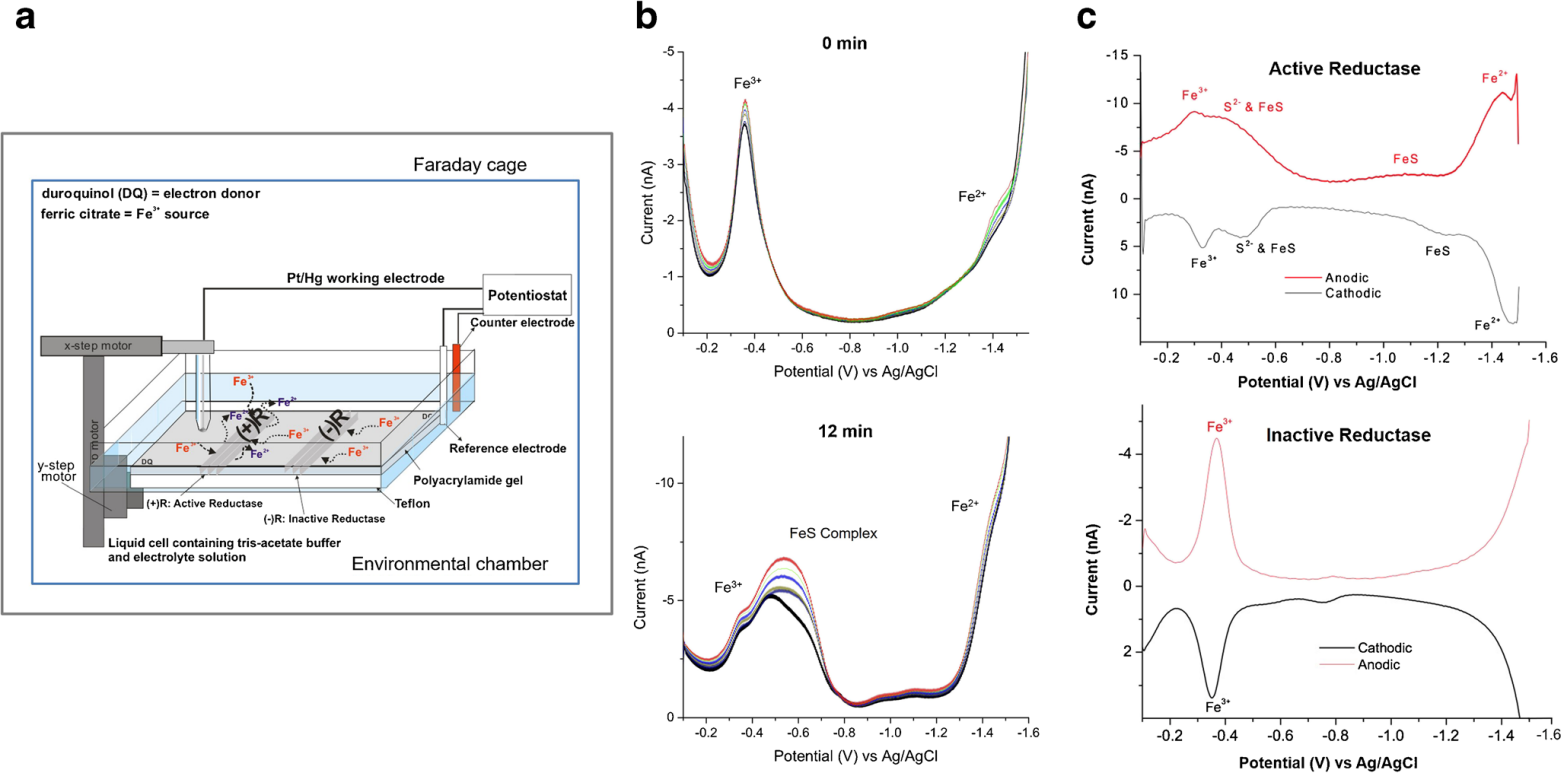
(**a**) Scheme of the SECM experimental setup within the environmental chamber controlling the O_2_ content below 2.5 ppm. (**b**) Square-wave voltammograms recorded at a Pt/Hg microelectrode positioned at 36 μm above the active reductase complexes (top) start of measurements at 0 min and (bottom) after 12 min. (**c**) Square-wave voltammograms recorded (top) above the redox-active protein and (bottom) above the inactive protein. Reprinted from Rudolph et al. [109], with permission from Elsevier

An example of SECM studies of anaerobic bacteria has been shown by Rudolph et al. [109]. The activity of Fe(III) and Mn(IV)–reducing proteins isolated from the outer membrane of *Shewanella oneidensis* was investigated. The extracted metal reductase complexes were separated by gel electrophoresis prior to SECM studies (schematically shown in Fig. 6a), which were performed in combination with square-wave anodic stripping voltammetry (SWASV) at Hg/Pt microelectrodes. Changes in concentration of iron(III) and the evolvement of a peak associated to sulphur species were monitored over time (Fig. 6b). Moreover, square-wave voltammograms above active and inactive reductase proteins confirmed the evidence of the enzymatic process, since iron(II) signal above the inactive enzyme was absent, as shown in Fig. 6c.

### Antibacterial agents

Forming biofilms provides protection to bacteria and ensures tolerance against antimicrobial treatments in comparison to their planktonic form. In recent years, strong efforts have been dedicated to the development of antimicrobial measures as biofilms are quite difficult to eradicate. In clinical scenarios, multiresistant bacterial strains present a significant threat as 75% of infections are related to biofilm formation. Quorum quenching enzymes [110], adhesin repressors [111], nanoparticle-based copolymers [56, 112] and metal-chelator approaches [113, 114] have been studied so far to prevent biofilm formation or to eradicate biofilms. To date, only a few studies concerning antimicrobial effects have been published using SECM [82, 112, 115, 116]. Silver nanoparticles (AgNPs) and Ag(I) at low concentrations are known as a very powerful antimicrobial agent, which, to date, are used in many consumer products and clinical applications [117]. Studies on the antibacterial mechanisms of Ag ions and AgNPs (smaller < 10 nm) showed that e.g. Ag ions react with peptidoglycans, resulting in cell membrane damage. Ag ions can also penetrate the cells, inducing increased levels of ROS and disruption of DNA replication cycles. The full mechanism of Ag(I)-induced cell death is not yet fully understood [118]. Holt and Bard [82] studied the influence of Ag ion concentration in respect to its toxicity using microelectrochemical and SECM experiments to measure oxygen and Ag(I) uptake by *E. coli* under different experimental conditions. Using a Clark microelectrode, the change of oxygen concentration over *E. coli* cells was monitored over time at different concentrations of AgNO_3_ in the presence of glucose. With these experiments, the authors showed that at a concentration of Ag(I) ≤ 10 mM, a stimulation of the bacterial respiration is triggered before cell death starts. Comparing this data with experiments performed with ferricyanide as an electron acceptor, the authors concluded that Ag(I) may block the electron flow in the electron transport chain breaking the respiratory chain.

**Fig. 7:**
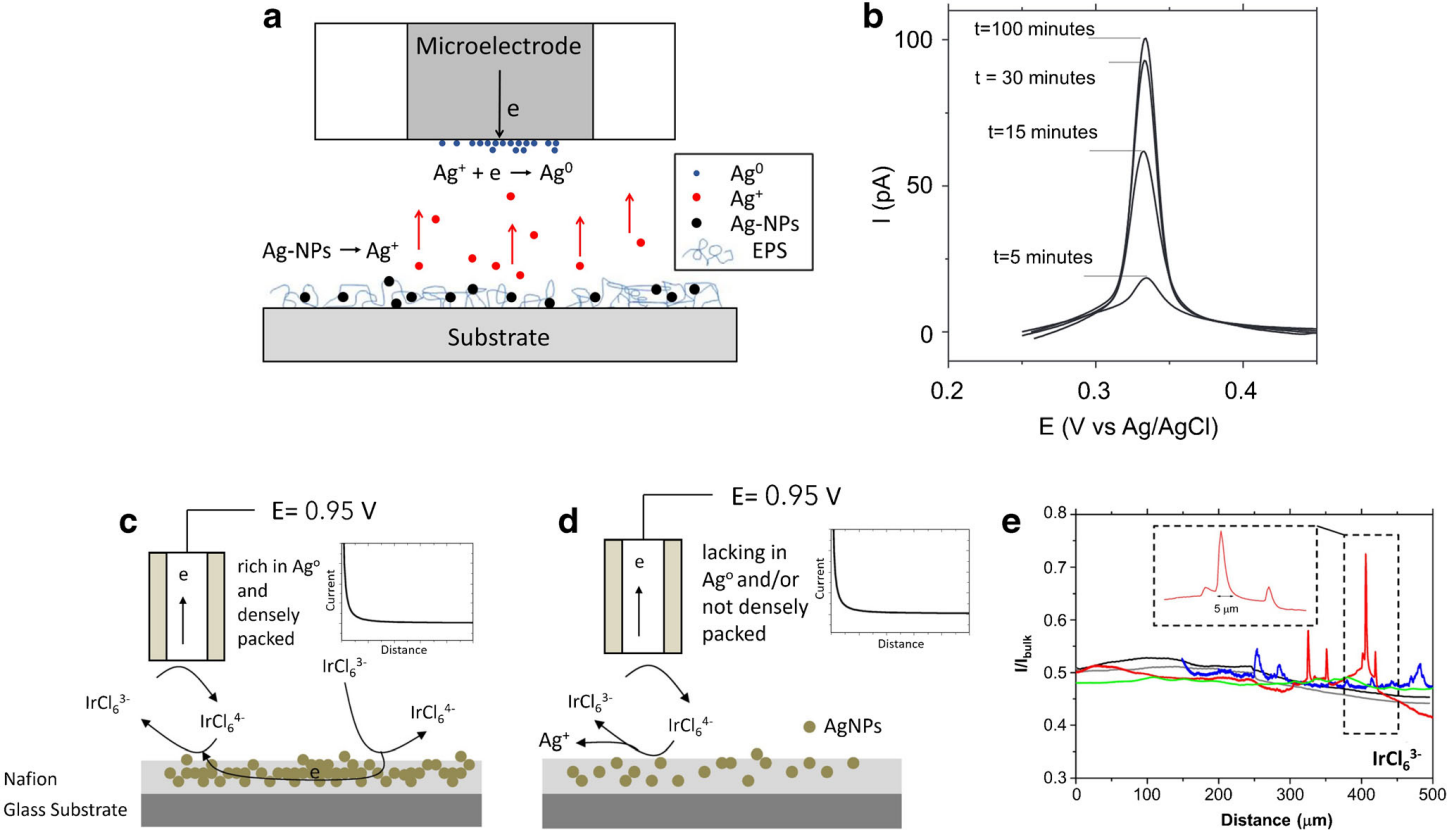
(**a**) Schematic of Ag(I) collection at the SECM tip. (**b**) Representative ASVs recorded above an AgNP-exopolysaccharide (EPS) surface over time. Reprinted from Battistel et al. [120], with permission from Elsevier. (**c**) Schematic of feedback mode experiments using [Ir(Cl)_6_]^3−^ as a redox mediator to study AgNP-rich surface. (**d**) Schematic of feedback mode experiments using [Ir(Cl)_6_]^3−^ as a redox mediator at a surface with low density of AgNPs. (**e**) SECM *x*-line scans recorded above the AgNP-Nafion surfaces using [Ir(Cl)_6_]^3−^ as a redox mediator. Reprinted from Pecchielan et al. [112], with permission from John Wiley and Sons

NPs, e.g. AgNPs embedded in polymeric matrices, allow a controlled release, which the degree of release can be tuned by the loading of NPs into the polymeric films [119]. The kinetics and temporal release behaviour of AgNPs and Ag ions can be studied locally via SECM, which provides in situ information on the oxidation state of the AgNPs embedded in the polymeric film [112]. Release studies of Ag(I) were performed via SECM in combination with anodic stripping voltammetry (ASV) [120], which enabled to detect selectively low concentrations of Ag(I). Oxidized Ag ions are first reduced (Ag^0^) at the surface of the Pt UME and then again stripped of (oxidized) as shown in Fig. 7a. Accumulation over time is shown in Fig. 7b, where oxidation current is proportional to the concentration of Ag(I). Alternatively, the reaction shown in Eq. (1) can be used to determine the Ag concentration

1 $$ {\left[\mathrm{Ir}{\left(\mathrm{Cl}\right)}_6\right]}^{2-}+{\mathrm{Ag}}^0\to {\left[\mathrm{Ir}{\left(\mathrm{Cl}\right)}_6\right]}^{3-}+{\mathrm{Ag}}^{+} $$

Feedback mode SECM experiments, as shown in Fig. 7c and d can be used to determine the electrochemical nature of the AgNPs using [Ir(Cl)_6_]^3−^ as a redox mediator. The regeneration of the redox mediator occurs only in the presence of Ag^0^ (positive feedback); however, in the absence or low density of AgNPs, the redox species is not re-generated, and a negative feedback is recorded. Pecchielan et al. [112] demonstrated such Ag release studies at Nafion films loaded with AgNPs (as shown in Fig. 7e).

Although to date only a limited number of papers have been published on the utility of SECM in bacterial research, within recent years, interest sparked due to the versatility of the technique and the improvements in hardware and imaging modes for studying life cells [121]. Table 1 presents an overview on studies conducted in the last decade using SECM and combined SECM techniques to investigate biofilms and antimicrobial agents.

**Table 1:** Applications of SECM for biofilm studies of bacterial and microbial cultures showing the examined species, the detected analyte and the used SECM probe

UME	Family of bacteria	Species detected	Reference
Platinum	*Pseudomonas aeruginosa*	PYO	[92, 93]
Pt-MWCNT-IL-GOD^a^	*Streptococcus mutans*	Glucose	[108]
Platinum	*Escherichia coli*	*p*-Aminophenol	[98]
Platinum	*Escherichia coli*	[Fe(CN)_6_]^3−/4−^	[82, 97]
Platinum	*Vibrio fischeri*	H_2_O_2_	[94]
Gold	*S. gordonii* and *A. actinomycetemcomitans*	H_2_O_2_	[81]
Pt-MWCNT-IL^b^	*S. gordonii*	H_2_O_2_	[95]
Clark UME	*Escherichia coli*	O_2_	[82]
Ca^2+^ microsensor	*Sporosarcina pasteurii*	Ca^2+^	[107]
Platinum	*Escherichia coli*	Cu^2+^	[122]
Pt/Hg UME	*Shewanella oneidensis*	Mn^2+^, Fe^2+^, S^2−^	[109]
Platinum-polyaniline	*Sporosarcina pasteurii*	pH	[107]
pH sensor	*S. gordonii* and *S. mutans*	pH	[88]
Platinum disk-silver ring	*Escherichia coli*	Ag^+^	[82]
Platinum	Antimicrobial surface	Ag^+^	[112]
Platinum	*Shewanella oneidensis*	H^+^	[123]

*UME* ultramicroelectrode

^a^Multiwalled carbon nanotubes in ionic liquid electrode functionalized with glucose oxidase

^b^Multiwalled carbon nanotubes in ionic liquid electrode

## Future potential of hybrid SECM techniques

In recent years, significant efforts have been dedicated to not only instrumental developments, for example incubator cells, which allow controlling the environment of the biological samples, but also hybrid SECM techniques, such as combining SECM with fluorescence microscopy for life sciences [124, 125] or hybrid scanning probe microscopy (SPM) methods taking advantage of SECM such as combined SICM-SECM [126] or AFM-SECM [127, 128]. Although such hybrid techniques have not yet been fully explored for studying bacteria, biofilms or antimicrobial coatings, in the future, they might have significant potential for improved lateral resolution along with correlating topographical changes to metabolic activity of biofilms. Also, combining adhesion measurements with mapping molecules appears highly attractive for in-depth understanding of the first steps of bacterial adhesion.

**Fig. 8:**
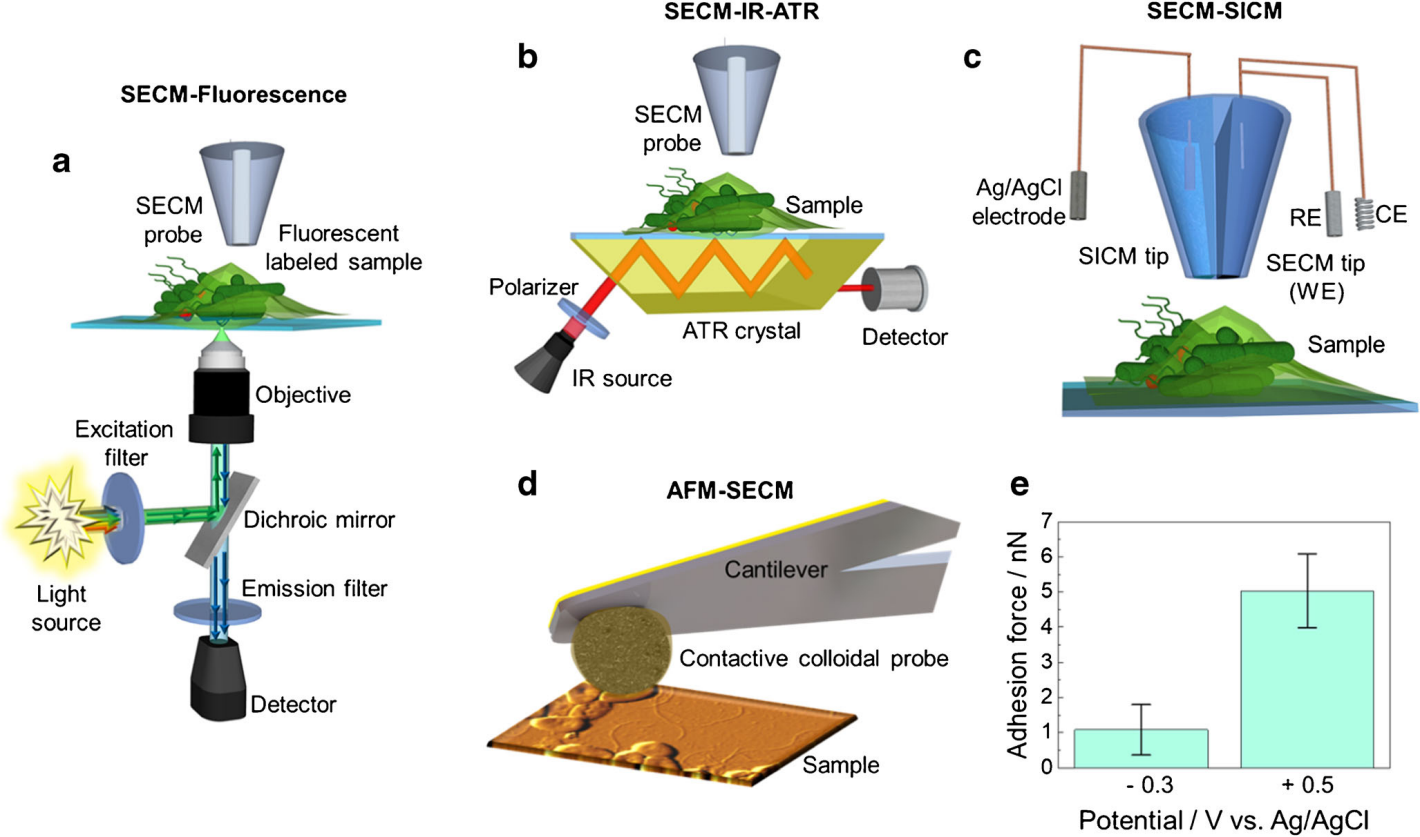
Schemes of SECM hybrid techniques. (**a**) SECM combined with fluorescence microscopy. (**b**) SECM in combination with IR-ATR. (**c**) SICM-SECM. (**d**) Colloidal AFM-SECM. (**e**) Adhesion measurements at the single bacterial level recorded with a biased colloidal AFM-SECM probe

Fluorescence microscopy has been intensively used to study biofilms and can be easily combined with SECM for life science studies. Cannan et al. [129], for example, used a microelectrode to change locally the pH via reduction of benzoquinone (BQ) to hydroquinone (HQ), a reaction that alkalizes the medium. As BQ is a fluorescent molecule and its concentration is pH-dependent, the 3D images obtained by CLSM correspond to the pH gradient adjacent to the electrode surface and are a function of the applied electrode potential. The combination of fluorescence with SECM has allowed researchers to study simultaneously the behaviour of small electroactive and fluorescent molecules which play a significant role in cellular processes such as ROS. Salamifar and Lai [125] monitored intracellular and extracellular ROS levels of prostate cancer cells using hybrid SECM-fluorescence microscopy, as shown schematically in Fig. 8a. An approach that is highly interesting to study biofilms as H_2_O_2_ or pH could be studied within and in close proximity at the biofilm. Electrochemical fluorophores, such as resazurin and tetrazines, used as respiration indicators to study bacteria and biofilm behaviour [130], have been tested as redox mediators. Guerret-Legras et al. [131, 132] showed that the fluorescence intensity of these molecules is a function of the potential of the SECM probe, independent of the substrate potential with the tip-substrate distance controlling the fluorescence amplitude. Hence, electrochemically induced processes in biological and microbial samples could be investigated with high spatial resolution.

Among the hybrid SPM techniques, the combination of SECM and SICM (Fig. 8c) will have advantages for studying biofilms, as this hybrid technique is truly non-invasive and significantly improves not only the achievable resolution as nanoelectrodes can be fabricated through carbon deposition methods inside the nanopipette, but it also allows to control the tip-substrate distance. This hybrid technique has been applied to investigate the local cell membrane permeability of electroactive species in myocyte cells [133] and cellular uptake at *Zea mays* root hair cells [134], but it has not yet been used for studying biofilms. However, the demonstrated highly localized uptake could contribute to understanding the efficacy in drug uptake or antimicrobial agents. The possibility to combine SECM with AFM has been demonstrated during the last two decades. AFM-SECM (Fig. 8d) allows, next to electrochemical imaging, simultaneously recording physical and high-resolution morphological information of the sample. AFM-SECM has been used to study biologically/biomedically relevant problems, i.e. mapping the flux of biomedically relevant electroactive molecules [128], detection of single-protein molecules at isolated viruses [127, 135] and mapping adhesion properties of mouse fibroblasts using conductive polymer–modified colloidal AFM-SECM probes [136]. Recently, Daboss et al. [137] investigated the adhesion of *Pseudomonas fluorescens* at the single bacterial cell level using polydopamine (PDA)-modified colloidal AFM-SECM probes. The functional groups of PDA can be selectively switched via the applied potential of the AFM-SECM probe, changing the adhesion forces due to the change in surface charges (as shown Fig. 8e). Combining SPM techniques with spectroscopic methods, for example combining SECM with IR-ATR (Fig. 8b) or Raman spectroscopy, is highly attractive to provide, next to electrochemical information, e.g. mapping respiratory activity and pH, also molecular-specific information on changes of the biofilm matrix. In general, hybrid methods have significant advantages as complementary information may be obtained simultaneously, eliminating uncertainties when interpreting separately albeit consecutively recorded information on complex samples such as biofilms. For example, combining SECM with confocal fluorescence microscopy would allow to map the changes in pH inside as well as pH changes in the local bacterial microenvironment at the same sample.

## Conclusions and future perspectives

This review highlights applications and potential of SECM for understanding key aspects in biofilm formation, monitoring metabolites and to elucidate quorum sensing/quenching processes, as well as to investigate antimicrobial effects. Many studies on the chemical composition and occurrence/disappearance of molecules are still performed ex situ, yet SECM may have significant advantages as information is obtained in situ and with temporal and spatial resolution.

An application area, which was not discussed in this review, is microbiologically induced corrosion (MIC), which is estimated to be a major cause for corrosion damage of metals, and is currently, predominantly studied using conventional electrochemical techniques. The ability of bacteria to use metals as electron donor in their respiration processes promotes the anodic dissolution of the metals with a corrosion rate, which is much higher than that in the absence of bacteria. As biofilms cause localized corrosion, SECM is a very suitable method to study processes including pit formation [138]. Hence, SECM may contribute to elucidate the role of bacteria and processes involved in biocorrosion and improve anticorrosive and antibiofilm treatments.

In particular, hybrid methods may allow in the future to contribute significantly to studies in heterogenous microbial systems. Considering the recently made instrumental improvements/developments in hyphenated methods, in particular in SPM, one can state: “there is plenty of room at the bottom,” which may, in the future, benefit microbial research. However, due to the complexity of biofilms, there is also a need for well-designed control experiments and a sufficient number of experiments to obtain meaningful statistical data, which might be sometimes challenging with these advanced high-resolution SPM methods.

Another interesting aspect to enhance SECM studies on complex samples such as biofilms in the future may be related to integrated machine learning approaches to perform automated SECM experiments. Barforoush et al. [139] implemented fuzzy logic algorithm for an automated approach of the SECM tip at different substrates (conductive and insulating) in addition to an automated tip/substrate alignment protocol. Screening approaches for photocatalyst and electrocatalyst have been demonstrated via SECM, although automated workflows and advanced robotics as demonstrated in i.e. materials science [140] have not yet been demonstrated for SECM.
